# Small-molecule screening of PC3 prostate cancer cells identifies tilorone dihydrochloride to selectively inhibit cell growth based on cyclin-dependent kinase 5 expression

**DOI:** 10.3892/or.2014.3174

**Published:** 2014-05-13

**Authors:** MICHEL D. WISSING, TIKVA DADON, EUNICE KIM, KLAUS B. PIONTEK, JOONG S. SHIM, NADINE S. KAELBER, JUN O. LIU, SUSHANT K. KACHHAP, BARRY D. NELKIN

**Affiliations:** 1 Department of Oncology, Sidney Kimmel Comprehensive Cancer Center, Johns Hopkins University School of Medicine, Baltimore, MD 21287, USA; 2 Department of Medical Oncology, Leiden University Medical Center, Leiden 2333ZA, The Netherlands; 3 Department of Pathology, University Medical Center Utrecht, Utrecht 3584CX, The Netherlands; 4 Department of Pharmacology and Molecular Sciences, Johns Hopkins University School of Medicine, Baltimore, MD 21205, USA

**Keywords:** tilorone, synthetic lethality, cyclin-dependent kinase 5, prostate cancer

## Abstract

Cyclin-dependent kinase 5 (CDK5) is a potential target for prostate cancer treatment, the enzyme being essential for prostate tumor growth and formation of metastases. In the present study, we identified agents that target prostate cancer cells based on CDK5 expression. CDK5 activity was suppressed by transfection of PC3 prostate cancer cells with a dominant-negative construct (PC3 CDK5dn). PC3 CDK5dn and PC3 control cells were screened for compounds that selectively target cells based on CDK5 expression, utilizing the Johns Hopkins Drug Library. MTS proliferation, clonogenic and 3D growth assays were performed to validate the selected hits. Screening of 3,360 compounds identified rutilantin, ethacridine lactate and cetalkonium chloride as compounds that selectively target PC3 control cells and a tilorone analog as a selective inhibitor of PC3 CDK5dn cells. A PubMed literature study indicated that tilorone may have clinical use in patients. Validation experiments confirmed that tilorone treatment resulted in decreased PC3 cell growth and invasion; PC3 cells with inactive CDK5 were inhibited more effectively. Future studies are needed to unravel the mechanism of action of tilorone in CDK5 deficient prostate cancer cells and to test combination therapies with tilorone and a CDK5 inhibitor for its potential use in clinical practice.

## Introduction

Although novel therapies have recently been introduced into clinical practice for the treatment of advanced prostate cancer, prostate cancer has remained the second deadliest cancer in men in the United States in 2014 (1). New therapeutic targets and strategies are urgently needed to further improve the clinical outcome of patients with prostate cancer.

One promising potential therapeutic target is cyclin-dependent kinase 5 (CDK5). CDK5 is a serine/threonine kinase structurally similar to other CDKs (2). CDK5 does not appear to have a major role in cell cycle regulation (3,4). It has been well characterized for its dominant role in the development of the central nervous system, including roles in neuronal migration, differentiation and adhesion (5,6). We and others subsequently showed that CDK5 plays an important role in cancer development and metastasis (7–12). In prostate cancer cells, we demonstrated that CDK5 was critical for cytoskeletal integrity, cell migration and invasion, and *in vivo*, for metastasis (7). In pancreatic cancer, CDK5 is intrinsic to KRAS signaling through the centrally important RAL signal transduction pathway, thus providing a potential ‘druggable’ target for mutant KRAS tumors (8). Together, these studies indicate that inhibition of CDK5, alone or in combination with other agents, may provide an effective therapeutic strategy for these and other cancer types.

In the present study we set out to identify agents that would be particularly effective in combination with CDK5 inhibition in prostate cancer cells. Therefore, we performed a screen of the Johns Hopkins Drug Library (JHDL). The JHDL is a collection of 3,360 pharmaceutical compounds that have successfully completed safety testing in humans for a variety of applications (13,14). This library has been used successfully for repurposing of compounds for cancer therapy, including identification of digoxin as an HIF1α inhibitor (15), and itraconazole as an angiogenesis inhibitor (16). We previously employed the JHDL to identify cetrimonium bromide and irinotecan as compounds with increased antitumor activity against prostate cancer cells expressing low levels of the metastasis suppressor gene N-myc downregulated gene 1 (NDRG1) (17). Here, we performed a similar JHDL screening with prostate cancer cells which differ in CDK5 activity. Tilorone was identified as a compound with *in vitro* synthetical lethality in CDK5-deficient prostate cancer cells.

## Materials and methods

### Cell culture

PC3 prostate cancer cell lines were obtained from ATCC. These cells are derived from a bone metastasis from a 62-year old prostate cancer patient. Human prostate fibroblasts, kindly provided by Dr J. Isaacs, were obtained from a prostate biopsy on a 62-year old prostate cancer patient with a Gleason score of 4. Both cell lines were grown and maintained in RPMI-1640 (Invitrogen) media supplemented with 10% fetal bovine serum. Cells were cultured in a humidified incubator at 37°C in a 5% CO_2_ atmosphere.

### Creation of the PC3 CDK5dn cell line

Loss of CDK5 function was accomplished in PC3 cells by transfection of a dominant-negative construct containing a D144N mutation, kindly provided by Dr L.H. Tsai (Harvard Medical School) (18). The protocol used has been described previously (7). In brief, the construct was subcloned in a bidirectional Tet vector, pBI-EGFP (BD Biosciences), which had a zeocin resistance gene added for selection (kindly provided by Dr K. Schuebel, Johns Hopkins University School of Medicine). pBI-EGFP empty vector or pBI-EGFP CDK5dn vector was transfected into PC3 cells which contained a Tet-Off promoter construct, pTTa (BD Biosciences).

### Western blotting

Western blotting was performed as described previously (19). Ten micrograms of protein was loaded on the gel. Primary antibodies were dissolved in blocking buffer [5% milk in TBST (100 mM Tris-HCl pH 7.4, 0.1% Tween-20, 150 mM NaCl in H_2_O)]. A 1:1,000 dilution was used for anti- CDK5 (Sigma-Aldrich); anti-vinculin (Millipore, Upstate) was diluted 1:4,000. Secondary antibodies were diluted at a 1:4,000 dilution. Normalization of the band intensity was carried out with the housekeeper protein vinculin. Developed blots were scanned using a Microtek scanner.

### Wound healing assay

Wound healing assays were performed with confluent PC3 control (containing the empty pBI-EGFP vector) or PC3 CDK5dn cells. A rubber-tipped scraper was used to scrape off an area of cells. Light microscopic images were captured immediately and 24 h after scraping.

### Small-molecule library screening

The JHDL library has been described previously (13,14,17). Storage and screening of JHDL compounds were carried out as described previously (17). Briefly, PC3 control and CDK5dn cells were seeded in 96-well plates (1×10^3^ cells/well) and allowed to adhere overnight. Then 5 μl of drugs, stored as stock solutions of 200 μM in DMSO/H_2_O, was added to complete RPMI media, so that cells were treated at a final concentration of 10 μM. After 48 h of treatment, 20 μl of MTS reagent from the CellTiter 96™ Aqueous Non-Radioactive Cell Proliferation Assay [a reagent containing 3-(4,5-dimethylthiazol-2-yl)-5-(3-carboxymethoxyphenyl)-2-(4-sulfophenyl)-2H-tetrazolium (MTS) and phenazine methosulfate (PMS); Promega] was added to each well for a duration of 2–4 h at 37°C. Plates were analyzed using a SoftMax Pro plate reader (Molecular Devices). Proliferation of treated cells was compared with proliferation of DMSO-treated PC3 control or CDK5dn cells (proliferation index). Proliferation indices of PC3 CDK5dn cells were compared to the proliferation indices of PC3 control cells. A PubMed study was performed to assess the clinical use of potential hits.

### MTS assays

MTS assays were performed to measure the antiproliferative effect of tilorone treatment. Tilorone dihydrochloride (Sigma-Aldrich) was stored as a 10 mM stock solution in DMSO at −20°C. One thousand PC3 cells were plated in 96-well plates containing 100 μl complete RPMI media. At circa 50% confluence, tilorone dihydrochloride was administered. For experiments the compound was diluted in complete RPMI media to obtain the desired final concentration. After treatment for 72 h (tilorone monotherapy), MTS reagent was added, and absorption at 490 nm was determined using a SoftMax Pro plate reader. Proliferation indices were calculated; untreated PC3 control or CDK5dn cells (in 103 μl complete RPMI media) were used as a control. Student’s t-tests were performed to assess p-values.

### Clonogenic assays

Clonogenic assays were performed to assess long-term survival after tilorone treatment. Prostate cancer cells were plated in 60 mm dishes and allowed to adhere. At 50–60% confluency, cells were treated with tilorone for 72 h. Subsequently, 1×10^3^ cells from each dish were plated in triplicate in 60-mm dishes and incubated in complete RPMI media for 12 days. Colonies were fixed and stained with a solution containing 90% methanol and 10% crystal violet solution (2.3% crystal violet, 0.1% ammonium oxalate and 20% ethyl alcohol; Sigma). Colonies were scanned with a computer scanner (Microtek) and counted manually. Student’s t-tests were performed to evaluate whether differences between cell lines were statistically significant.

### 3D growth assay

3D growth assays were performed utilizing the same protocol as described previously (17). In short, spheroids were generated by culturing PC3 cells for 16 h as a hanging drop over a humidified plate in a CO_2_ incubator in complete RPMI media containing 0.5% methylcellulose. Spheroids were embedded in collagen matrix (BD Biosciences), treated with tilorone, and imaged using a Nikon Eclipse Ti microscope (Nikon) on the day of treatment and six days after treatment start. Spheroid and total (spheroid plus sprouts) areas were measured with ImageJ. Fold increases were calculated by dividing the spheroid/total area at day 6 by the spheroid/total area on day 0 for each individual spheroid. For each cell line and time point, fold increases of four spheroids were averaged. Statistical analyses were performed using Student’s t-tests.

## Results

### Suppression of CDK5 activity

**Figure 1:**
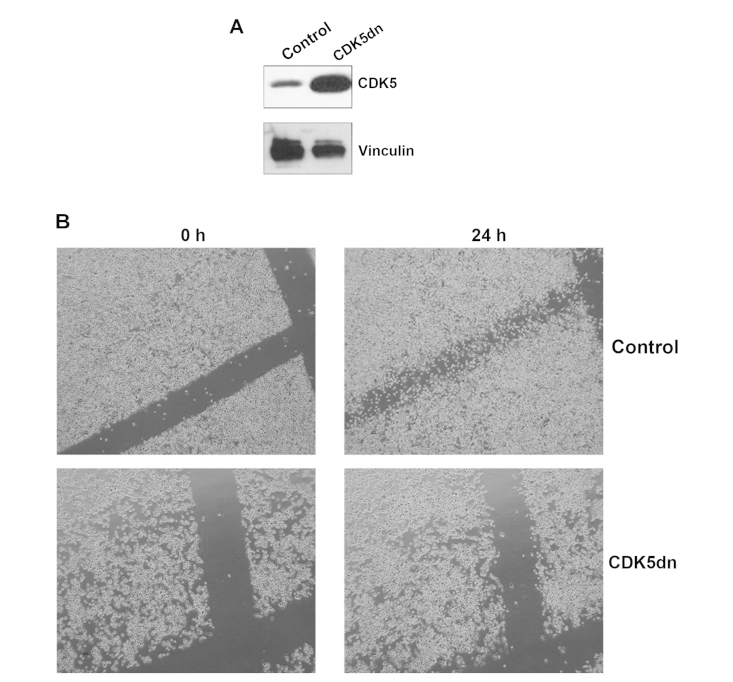
Suppression of CDK5 activity by transfection of a dominant-negative construct. (A) PC3 cells were transfected with a pBI-EGFP vector with or without a CDK5 dominant-negative construct; clones in which transfection was successful were selected. A western blotting for CDK5 was performed with these PC3 CDK5dn and control cell lysates. (B) Wound healing assay with PC3 CDK5dn and control cells to confirm CDK5 inactivity in PC3 CDK5dn cells. CDK5, cyclin-dependent kinase 5.

PC3 prostate cancer cells were chosen for the JHDL compound screen due to their highly metastatic potential and androgen independence, thereby resembling aggressive metastatic castrate-resistant prostate cancer. CDK5 activity was inhibited by transfection and selection of a dominant-negative mutation (CDK5 144N). These PC3 CDK5dn cells had a higher protein level of total CDK5 as compared to the PC3 control cells (PC3 cells transfected with an empty vector) (Fig. 1A). A wound healing assay (8) confirmed that CDK5 was functionally inactive in these cells; unlike the PC3 control cells, PC3 CDK5dn cells did not have the ability to invade the scraped surface area (Fig. 1B).

### Library screen for compounds targeting PC3 cells based on CDK5 activity

**Figure 2:**
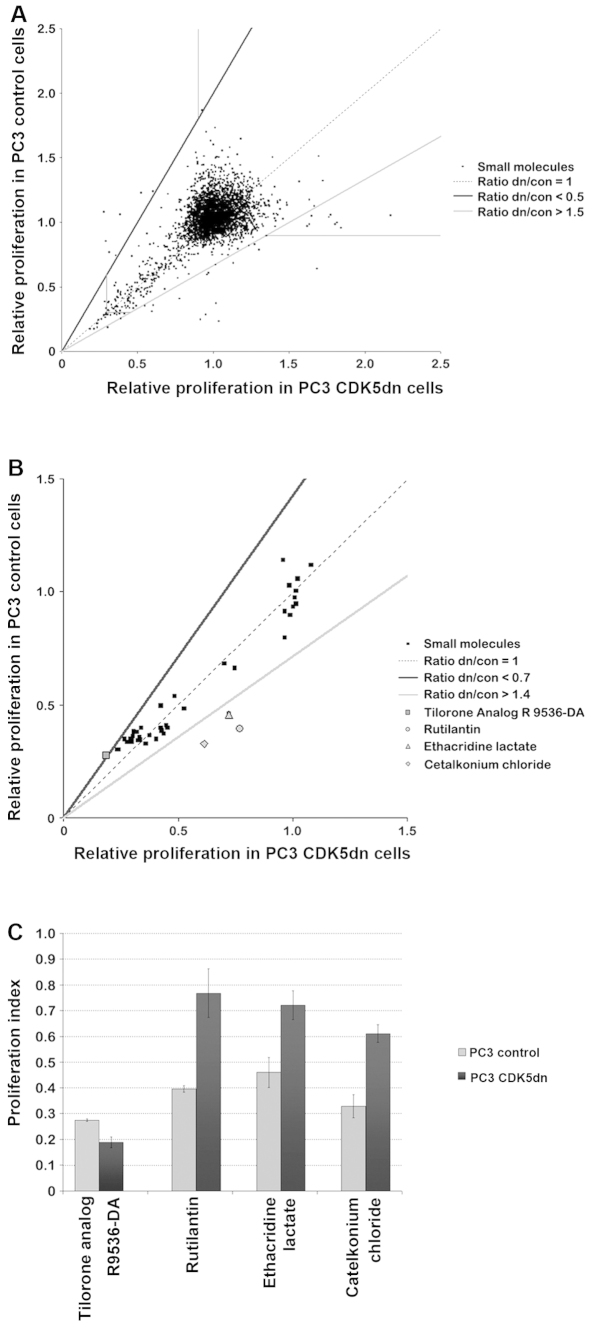
Screening of the JHDL identifies four small molecules that target PC3 cells selectively based on CDK5 activity. (A) Primary screen with all 3,360 compounds of the JHDL, by performing MTS assays after 48 h of treatment with the compounds at 10 μM. Compounds were selected when the proliferation index ratio CDK5dn/control was below 0.5 or above 1.5. Compounds were excluded as a hit when not resulting in a decreased cell proliferation of 10% in one of the cell lines compared to DMSO-treated controls. Compounds inhibiting cell proliferation by >70% were also selected. (B) Secondary screen by performing MTS assays in triplicate with PC3 cells treated with 41 selected compounds from the primary screen. Compounds were selected when the proliferation index ratio CDK5dn/control was below 0.7 or above 1.4. (C) Four compounds selected from the secondary screen. Tilorone was highly effective against PC3 cells, particularly PC3 CDK5dn cells. The other three compounds primarily targeted PC3 cells with active CDK5. Error bars display the standard deviation of three independent measurements. CDK5, cyclin-dependent kinase 5; JHDL, Johns Hopkins Drug Library.

A high-throughput screening assay was performed to select compounds that target PC3 cells based on CDK5 activity. PC3 control and CDK5dn cells were treated with all compounds of the JHDL at 10 μM for 48 h. To identify hits that selectively target PC3 cells based on CDK5 expression, we selected all compounds in which the proliferation index ratio (CDK5dn/control) was below 0.5 or above 1.5 (Fig. 2A). Furthermore, hits had to inhibit cell proliferation of PC3 cells by at least 10%, as we were specifically interested in compounds that inhibited cell growth (horizontal and vertical line in graph). We also selected all compounds that inhibited cell proliferation in PC3 cells by 70% (bottom left corner of the graph), as we were interested in identifying potential highly effective antitumor agents. In total, 41 hits were selected for further evaluation.

A secondary screen was performed in which selected hits from the primary screen were added at 10 μM for 48 h to PC3 control and CDK5dn cells in triplicate, to weed out false positive results (Fig. 2B). Cutoff values were slightly less strict than in the primary screen; compounds were considered a hit when the ratio of proliferation indices (CDK5dn/control) was below 0.7 or above 1.4. This resulted in the identification of three compounds that selectively target CDK5-expressing PC3 cells: rutilantin, ethacridine lactate and cetalkonium chloride (Fig. 2C). These compounds have not been used as antitumor agents and their potential clinical use as intravenous antitumor agents seems limited (20–23). Another compound, tilorone analog R9536-DA, was highly effective in inhibiting both isogenic PC3 cell lines (>70% inhibition), but it inhibited proliferation of PC3 CDK5dn cells somewhat more effectively (ratio CDK5dn/control: 0.687). Tilorone and its analogs have antiviral activity, acting at least in part as interferon inducers (24–26) and have been shown preclinically and clinically to have antitumor activity as well (27,28).

### Tilorone selectively targets PC3 cells with low CDK5 activity

**Figure 3:**
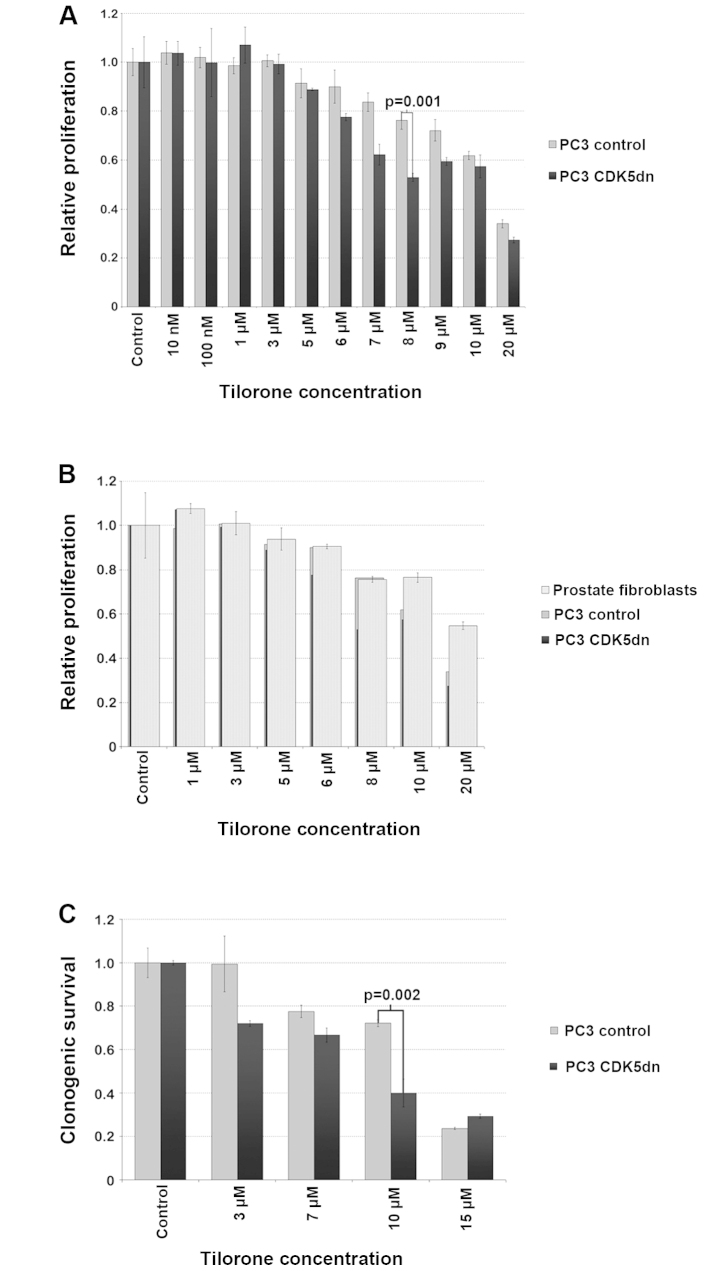
Validation of tilorone dihydrochloride as a compound that selectively targets PC3 cells based on CDK5 activity. (A) MTS assays performed after treating PC3 CDK5dn and control cells for 72 h with tilorone. (B) MTS assays performed after 72 h treatment of healthy human prostate fibroblasts with tilorone to determine toxicity of tilorone in healthy cells. (C) Clonogenic survival assays performed after a 72-h treatment of PC3 cells with tilorone, further validating the screening results. Error bars display the standard deviation from at least three independent measurements. CDK5, cyclin-dependent kinase 5.

We continued our experiments with freshly dissolved tilorone dihydrochloride. After 72 h of tilorone treatment at various concentrations, its IC_50_ was established at 8–12 μM in PC3 CDK5dn cells and 15 μM in PC3 control cells in MTS assays (Fig. 3A). At 8 μM, proliferation activity was decreased by 24 and 47% in the PC3 control and CDK5dn cells, respectively (p=0.001). To assess toxicity of tilorone in normal prostate cells, MTS assays were performed with tilorone treatment of human prostate fibroblasts (Fig. 3B). Sensitivity of these cells to tilorone was similar to that of the PC3 control cells.

The inhibitory effect of tilorone in PC3 cells was further assessed by performing clonogenic assays (Fig. 3C). PC3 CDK5dn cells were also significantly more sensitive than PC3 control cells to tilorone in this assay. Treatment with 10 μM tilorone resulted in clonogenic survival of 40% in PC3 CDK5dn cells and 72% in PC3 control cells (p=0.002).

**Figure 4:**
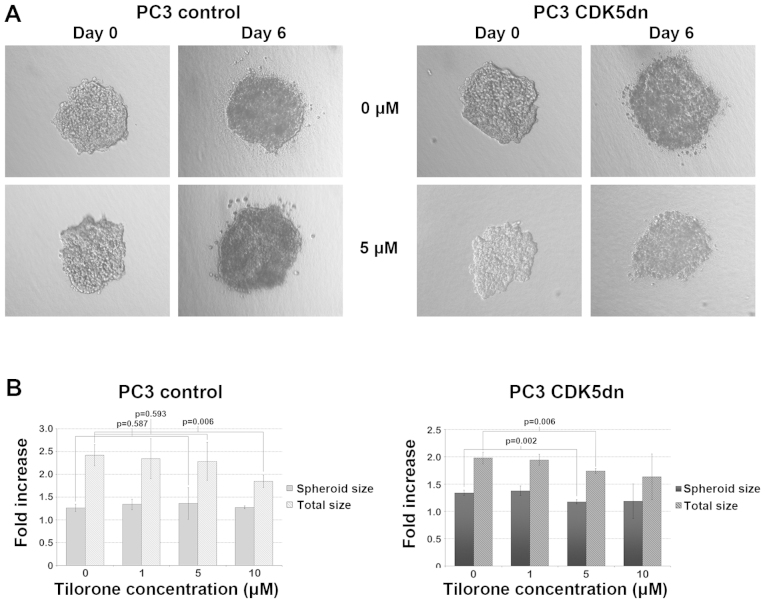
3D hanging drop growth assay performed to assess 3D spheroid growth and invasive potential of PC3 cells upon tilorone treatment. (A) Representative images of PC3 control (left) and CDK5dn (right) cells at day 0 and day 6, either untreated or treated with 5 μM tilorone. (B) Fold increase in spheroid and total size after six days of tilorone treatment at various concentrations. Error bars display the standard deviation of four independent measurements. CDK5, cyclin-dependent kinase 5.

A spheroid growth assay was performed to assess 3D tumor growth and invasion of PC3 cells upon tilorone treatment (Fig. 4). Both PC3 control and PC3 CDK5dn cells had comparable increases in spheroid size over six days. However, total size (the size of spheroids plus sprouts) had a higher fold increase in PC3 control cells, confirming that untreated PC3 CDK5dn cells had a decreased invasive potential as compared to PC3 control cells. When tilorone was administered at 5 μM, PC3 control spheroids had a similar growth and invasive pattern as the untreated PC3 control cells (p=0.59) (Fig. 4B, left graph). However, when tilorone was administered at the same concentration to PC3 CDK5dn cells, a significant decrease in both spheroid size and total size was observed (p<0.01), suggesting that tilorone successfully inhibits spheroid growth and invasion of PC3 CDK5dn cells when administered at 5 μM (Fig. 4B, right graph). At 10 μM, both isogenic cell lines had a decreased invasive potential.

## Discussion

The JHDL, a library of well characterized pharmaceutical compounds, was developed to facilitate drug repurposing studies (29). The extensive *in vivo* toxicity and pharmacokinetic profiles of compounds in the library allow rapid subsequent development of these compounds. Several compounds from the JHDL have been advanced to clinical trials for cancer and other therapeutic applications (13,14,16,17,30–32).

In the present study we screened the JHDL for compounds that differentially inhibit cancer cell growth in the presence of CDK5 inhibition; tilorone and a tilorone analog were identified as agents that selectively target CDK5-deficient PC3 prostate cancer cells. Tilorone (Amixin IC) is employed clinically in some countries as an orally active antiviral agent (25). Tilorone has been tested in humans for the treatment of cerebral gliomas, laryngeal papillomatosis and breast cancer (28,33,34). Although antitumor efficacy was reported, interest in tilorone for cancer therapy has subsided. Recently, Zhou *et al* reported new tilorone analogs with improved anticancer activity (35). These analogs may be promising to examine, particularly in combination with CDK5 inhibition.

In addition to the possibility that tilorone may be promising in combination with CDK5 inhibition, the identification of tilorone as an agent that selectively targets cells with inactive CDK5 suggests potential classes of drugs to potentiate the efficacy of CDK5 inhibition. Tilorone has been characterized as an interferon inducer (24). This suggests that interferon itself, or an alternative interferon inducer such as a TLR agonist, may be useful in combination with a CDK5 inhibitor. Nevertheless, other mechanisms may be involved. For example, tilorone is a DNA intercalating agent as well (24) and one may envision that it may modulate chromatin structure and gene expression. Other functions of tilorone, including signaling pathway and transcription factor interactions (36,37), may also be involved. Further studies are needed to unravel the exact mechanism of action by which tilorone selectively targets CDK5-negative prostate cancer cells.

## References

[1] Siegel R, Ma J, Zou Z, Jemal A (2014). Cancer statistics, 2014. CA Cancer J Clin.

[2] Tarricone C, Dhavan R, Peng J, Areces LB, Tsai LH, Musacchio A (2001). Structure and regulation of the CDK5-p25(nck5a) complex. Mol Cell.

[3] Rosales JL, Lee KY (2006). Extraneuronal roles of cyclin-dependent kinase 5. Bioessays.

[4] Lalioti V, Pulido D, Sandoval IV (2010). Cdk5, the multifunctional surveyor. Cell Cycle.

[5] Dhavan R, Tsai LH (2001). A decade of CDK5. Nat Rev Mol Cell Biol.

[6] Cicero S, Herrup K (2005). Cyclin-dependent kinase 5 is essential for neuronal cell cycle arrest and differentiation. J Neurosci.

[7] Strock CJ, Park JI, Nakakura EK, Bova GS, Isaacs JT, Ball DW, Nelkin BD (2006). Cyclin-dependent kinase 5 activity controls cell motility and metastatic potential of prostate cancer cells. Cancer Res.

[8] Feldmann G, Mishra A, Hong SM (2010). Inhibiting the cyclin-dependent kinase CDK5 blocks pancreatic cancer formation and progression through the suppression of Ras-Ral signaling. Cancer Res.

[9] Hsu FN, Chen MC, Chiang MC (2011). Regulation of androgen receptor and prostate cancer growth by cyclin-dependent kinase 5. J Biol Chem.

[10] Demelash A, Rudrabhatla P, Pant HC (2012). Achaete-scute homologue-1 (ASH1) stimulates migration of lung cancer cells through Cdk5/p35 pathway. Mol Biol Cell.

[11] Hsu FN, Chen MC, Lin KC (2013). Cyclin-dependent kinase 5 modulates STAT3 and androgen receptor activation through phosphorylation of Ser^727^on STAT3 in prostate cancer cells. Am J Physiol Endocrinol Metab.

[12] Pozo K, Castro-Rivera E, Tan C (2013). The role of Cdk5 in neuroendocrine thyroid cancer. Cancer Cell.

[13] Chong CR, Qian DZ, Pan F, Wei Y, Pili R, Sullivan DJ, Liu JO (2006). Identification of type 1 inosine monophosphate dehydrogenase as an antiangiogenic drug target. J Med Chem.

[14] Chong CR, Xu J, Lu J, Bhat S, Sullivan DJ, Liu JO (2007). Inhibition of angiogenesis by the antifungal drug itraconazole. ACS Chem Biol.

[15] Rudin CM, Liu W, Desai A (2008). Pharmacogenomic and pharmacokinetic determinants of erlotinib toxicity. J Clin Oncol.

[16] Zhang H, Qian DZ, Tan YS (2008). Digoxin and other cardiac glycosides inhibit HIF-1alpha synthesis and block tumor growth. Proc Natl Acad Sci USA.

[17] Wissing MD, Mendonca J, Kim E (2013). Identification of cetrimonium bromide and irinotecan as compounds with synthetic lethality against NDRG1 deficient prostate cancer cells. Cancer Biol Ther.

[18] Nikolic M, Dudek H, Kwon YT, Ramos YF, Tsai LH (1996). The cdk5/p35 kinase is essential for neurite outgrowth during neuronal differentiation. Genes Dev.

[19] Kachhap SK, Rosmus N, Collis SJ (2010). Downregulation of homologous recombination DNA repair genes by HDAC inhibition in prostate cancer is mediated through the E2F1 transcription factor. PLoS One.

[20] O’Meara S, Al-Kurdi D, Ologun Y, Ovington LG (2010). Antibiotics and antiseptics for venous leg ulcers. Cochrane Database Syst Rev.

[21] Hou SP, Fang AH, Chen QF, Huang YM, Chen OJ, Cheng LN (2011). Termination of second-trimester pregnancy by mifepristone combined with misoprostol versus intra-amniotic injection of ethacridine lactate (Rivanol^®^): a systematic review of Chinese trials. Contraception.

[22] Daull P, Lallemand F, Garrigue JS (2013). Benefits of cetalkonium chloride cationic oil-in-water nanoemulsions for topical ophthalmic drug delivery. J Pharm Pharmacol.

[23] Hume V, Westwood JC, Appleyard G (1965). The anti-viral action of Rutilantin A. J Gen Microbiol.

[24] Krueger RE, Mayer GD (1970). Tilorone hydrochloride: an orally active antiviral agent. Science.

[25] Mayer GD, Krueger RF (1970). Tilorone hydrochloride: mode of action. Science.

[26] Tazulakhova EB, Parshina OV, Guseva TS, Ershov FI (2001). Russian experience in screening, analysis, and clinical application of novel interferon inducers. J Interferon Cytokine Res.

[27] Adamson RH (1971). Antitumor activity of tilorone hydrochloride against some rodent tumors: preliminary report. J Natl Cancer Inst.

[28] Cummings FJ, Gelman R, Skeel RT, Kuperminc M, Israel L, Colsky J, Tormey D (1981). Phase II trials of Baker’s antifol, bleomycin, CCNU, streptozotocin, tilorone, and 5-fluorodeoxyuridine plus arabinosyl cytosine in metastatic breast cancer. Cancer.

[29] Chong CR, Sullivan DJ (2007). New uses for old drugs. Nature.

[30] Shim JS, Matsui Y, Bhat S (2010). Effect of nitroxoline on angiogenesis and growth of human bladder cancer. J Natl Cancer Inst.

[31] Shim JS, Rao R, Beebe K, Neckers L, Han I, Nahta R, Liu JO (2012). Selective inhibition of HER2-positive breast cancer cells by the HIV protease inhibitor nelfinavir. J Natl Cancer Inst.

[32] Yang HC, Xing S, Shan L (2009). Small-molecule screening using a human primary cell model of HIV latency identifies compounds that reverse latency without cellular activation. J Clin Invest.

[33] Lisianyi MI, Skitiak SA (2002). Use of amiksin in complex therapy of cerebral gliomas. Lik Sprava.

[34] Karimova FS, Ivanchenko GF, Grigorian SS (2000). The treatment of laryngeal papillomatosis with interferon inducers. Vestn Otorinolaringol.

[35] Zhou D, Tuo W, Hu H (2013). Synthesis and activity evaluation of tilorone analogs as potential anticancer agents. Eur J Med Chem.

[36] Ratan RR, Siddiq A, Aminova L (2008). Small molecule activation of adaptive gene expression: tilorone or its analogs are novel potent activators of hypoxia inducible factor-1 that provide prophylaxis against stroke and spinal cord injury. Ann NY Acad Sci.

[37] Schrimpf MR, Sippy KB, Briggs CA (2012). SAR of α7 nicotinic receptor agonists derived from tilorone: exploration of a novel nicotinic pharmacophore. Bioorg Med Chem Lett.

